# The impact of artificial intelligence on retinal disease management: Vision Academy retinal expert consensus

**DOI:** 10.1097/ICU.0000000000000980

**Published:** 2023-07-13

**Authors:** Carla Danese, Aditya U. Kale, Tariq Aslam, Paolo Lanzetta, Jane Barratt, Yu-Bai Chou, Bora Eldem, Nicole Eter, Richard Gale, Jean-François Korobelnik, Igor Kozak, Xiaorong Li, Xiaoxin Li, Anat Loewenstein, Paisan Ruamviboonsuk, Taiji Sakamoto, Daniel S.W. Ting, Peter van Wijngaarden, Sebastian M. Waldstein, David Wong, Lihteh Wu, Miguel A. Zapata, Javier Zarranz-Ventura

**Affiliations:** a Department of Medicine – Ophthalmology, University of Udine, Udine, Italy; b Department of Ophthalmology, AP-HP Hôpital Lariboisière, Université Paris Cité, Paris, France; c Academic Unit of Ophthalmology, Institute of Inflammation & Ageing, College of Medical and Dental Sciences, University of Birmingham, Birmingham; d Division of Pharmacy and Optometry, Faculty of Biology, Medicine and Health, University of Manchester School of Health Sciences, Manchester, UK; e International Federation on Ageing, Toronto, Canada; f Department of Ophthalmology, Taipei Veterans General Hospital; g School of Medicine, National Yang Ming Chiao Tung University, Taipei, Taiwan; h Department of Ophthalmology, Hacettepe University, Ankara, Turkey; i Department of Ophthalmology, University of Münster Medical Center, Münster, Germany; j Department of Ophthalmology, York Teaching Hospital NHS Foundation Trust, York, UK; k Service d’ophtalmologie, CHU Bordeaux; l University of Bordeaux, INSERM, BPH, UMR1219, F-33000 Bordeaux, France; m Moorfields Eye Hospital Centre, Abu Dhabi, UAE; n Istituto Europeo di Microchirurgia Oculare, Udine, Italy; o Tianjin Key Laboratory of Retinal Functions and Diseases, Tianjin Branch of National Clinical Research Center for Ocular Disease, Eye Institute and School of Optometry, Tianjin Medical University Eye Hospital, Tianjin; p Xiamen Eye Center, Xiamen University, Xiamen, China; q Division of Ophthalmology, Tel Aviv Sourasky Medical Center, Sackler Faculty of Medicine, Tel Aviv University, Tel Aviv, Israel; r Department of Ophthalmology, College of Medicine, Rangsit University, Rajavithi Hospital, Bangkok, Thailand; s Department of Ophthalmology, Kagoshima University, Kagoshima, Japan; t Singapore National Eye Center, Duke-NUS Medical School, Singapore; u Ophthalmology, Department of Surgery, University of Melbourne, Melbourne, Australia; v Centre for Eye Research Australia, Royal Victorian Eye and Ear Hospital, East Melbourne, Victoria, Australia; w Department of Ophthalmology, Landesklinikum Mistelbach-Gänserndorf, Mistelbach, Austria; x Unity Health Toronto – St. Michael's Hospital, University of Toronto, Toronto, Canada; y Macula, Vitreous and Retina Associates of Costa Rica, San José, Costa Rica; z Ophthalmology Department, Hospital Vall d’Hebron; aa Hospital Clínic de Barcelona, University of Barcelona, Barcelona, Spain

**Keywords:** artificial intelligence, disease management, retina

## Abstract

The aim of this review is to define the “state-of-the-art” in artificial intelligence (AI)-enabled devices that support the management of retinal conditions and to provide Vision Academy recommendations on the topic.

**Recent findings:**

Most of the AI models described in the literature have not been approved for disease management purposes by regulatory authorities. These new technologies are promising as they may be able to provide personalized treatments as well as a personalized risk score for various retinal diseases. However, several issues still need to be addressed, such as the lack of a common regulatory pathway and a lack of clarity regarding the applicability of AI-enabled medical devices in different populations.

**Summary:**

It is likely that current clinical practice will need to change following the application of AI-enabled medical devices. These devices are likely to have an impact on the management of retinal disease. However, a consensus needs to be reached to ensure they are safe and effective for the overall population.

## INTRODUCTION

Artificial intelligence (AI) is a branch of computer science, statistics, and engineering that uses algorithms or models to perform tasks that mimic human capabilities [1]. Machine learning is a subset of AI that allows computer algorithms to learn through data to perform a task without being explicitly programmed [1]. The value of digital technologies in contributing to advancing universal health coverage has been recognized. A consensus was reached on the need for more research on the acceptability, feasibility, and ethics regarding the use of AI for the development of decision support systems [2].

There is great interest in developing AI medical devices, especially in imaging-focused specialties such as ophthalmology [3]. At present, there is no consensus on the evidence requirements for AI medical devices in ophthalmology [3].

Most of the AI medical devices licensed for clinical application in ophthalmology by regulatory authorities such as the U.S. Food and Drug Administration and Conformité Européenne are approved for diagnostic purposes rather than for disease management. Chou and colleagues, on behalf of the Vision Academy, have developed an accompanying expert consensus paper on the use of AI medical devices in diagnosis and screening [4]. This paper will focus on the capabilities of AI in predicting functional and structural changes during retinal disease, as well as treatment outcomes of patients affected by retinal diseases. 

**Box 1:**
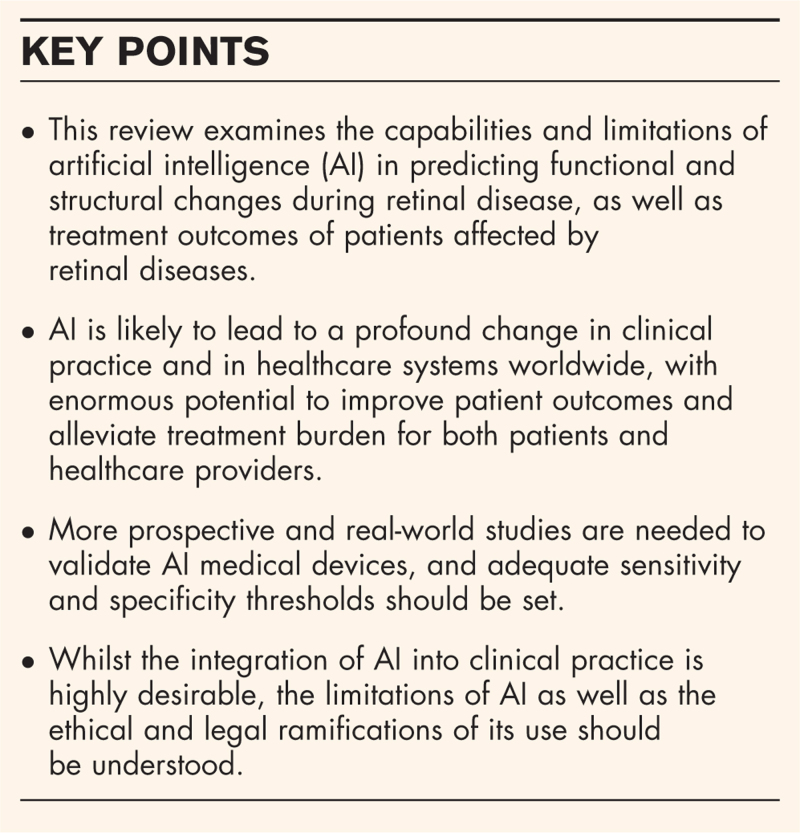
no caption available

## OVERVIEW OF APPROVED ARTIFICIAL INTELLIGENCE MEDICAL DEVICES FOR RETINAL DISEASE MANAGEMENT

Table 1 provides a list of approved AI medical devices with the potential to impact retinal disease management. It also details the search strategy used to identify the devices. Most of these devices are cloud-based.

**Table 1:** Summary of current AI systems with regulatory approval for retinal disease management

AI system	Company	Year approved	Regulatory authority	Exam analyzed	Target	Impact on prognosis	Cloud-based?
Retmarker DR Biomarker [5]	Retmarker, SA, Taveiro, Portugal	2010	CE, TGA (Australia)	Color fundus images	DR	Identification of the risk of complications	No
Notal Home OCT [6]	Notal Vision, Inc., Manassas, VA, USA	2018	FDA (Breakthrough Device Designation)	OCT	Neovascular AMD	Early detection of fluid	Yes
Notal OCT Analyzer [7]	Notal Vision, Inc., Manassas, VA, USA	2018	FDA (Breakthrough Device Designation)	OCT	Neovascular AMD, DME, RVO	Automated detection of fluid	Yes
RetinAI Discovery [8]	RetinAI Medical AG, Bern, Switzerland	2020	CE, FDA	OCT, color fundus images	AMD, DR, epiretinal membranes, other	Strict follow-up	Yes
VUNO Med-Fundus AI [9]	VUNO Inc., Seoul, Korea	2020	CE, MFDS (Korea), HAS (Singapore)	Color fundus images	AMD, DR, epiretinal membranes, other	Strict follow-up	No
iPredict [10]	iHealthScreen Inc., Richmond Hill, NY, USA	2021 and 2022	CE, TGA (Australia)	OCT, color fundus images	AMD, DR	Prediction of progression	Yes
RetInSight Fluid Monitor [11]	RetInSight GmbH, Vienna, Austria	2022	CE	OCT	AMD, DME	Automated detection of fluid	Yes

*Note:* All AI algorithms involved in the management of retinal diseases which had already received approval from the FDA, CE, or other regulatory authorities, or were undergoing the process of approval were included. These devices were searched for in the European Database on Medical Devices (EUDAMED) database [12], the FDA website (on the webpage of Artificial Intelligence and Machine Learning-Enabled Medical Devices) [13], and the FDA 510(k) Premarket Notification [14]. Algorithms that had received approval between January 2015 and December 2022 were included. We searched PubMed for available literature on the scientific evidence of the efficacy of these systems and for additional details regarding newer, unapproved algorithms. Only English-written articles published up to March 2023 were considered. AMD, age-related macular degeneration; CE, Conformité Européenne; DME, diabetic macular edema; DR, diabetic retinopathy; FDA, U.S. Food and Drug Administration; MFDS, Ministry of Food and Drug Safety (Korea); OCT, optical coherence tomography; RVO, retinal vein occlusion; TGA, Therapeutic Goods Administration.

RetinAI Discovery (RetinAI Medical AG, Bern, Switzerland) and VUNO Med-Fundus AI (VUNO Inc., Seoul, Korea) are designed to identify biomarkers in retinal diseases, such as diabetic retinopathy, age-related macular degeneration (AMD), and epiretinal membranes, thus providing effective support to physicians in disease management. While RetinAI Discovery works on optical coherence tomography (OCT) and color fundus images [15–17], VUNO Med-Fundus AI is applicable to color fundus images and also provides heatmaps of abnormal findings [18]. Heatmaps are a visual representation of regions of an image that influence the classification of that image by the AI medical devices [19]. Heatmaps can be applied to diagnosis and strict follow-up of the disease [18]. RetinAI Discovery is currently being evaluated in the RAZORBILL study, an observational, noninterventional, open-label study evaluating its influence on disease activity assessment in patients with neovascular AMD; it has already been evaluated in a multicenter, national, real-world cohort [20^▪^]. The RAZORBILL study will also evaluate the effect of AI on clinical workflow [21^▪▪^].

iPredict (iHealthScreen Inc., Richmond Hill, NY, USA) also assesses color fundus images and OCT. It is an autonomous system that can highlight abnormalities in a single image, as well as assess differences between two images taken at different times to evaluate retinal disease progression [10,22]. Similarly, Retmarker DR Biomarker (Retmarker Ltd, Coimbra, Portugal) aims to predict the risk of developing sight-threatening complications of diabetic retinopathy through the identification of microaneurysms on color fundus images [23].

The timely and personalized treatment of patients with neovascular AMD is a relevant issue in ophthalmology and is targeted by several AI medical devices. RetInSight Fluid Monitor (RetInSight GmbH, Vienna, Austria) quantifies intraretinal and subretinal fluid as well as subretinal pigment epithelial fluid on OCT images, thus providing support to physicians [24^▪^]. At-home devices to enable more frequent monitoring of patients with AMD may represent a revolution in the field. Notal Home OCT [6] and Notal OCT Analyzer [7] (Notal Vision Inc., Manassas, VA, USA) are capable of automated detection of retinal fluid, leading to early fluid detection and more intensive disease monitoring. The first is a home device used by patients already diagnosed with neovascular AMD [25,26], while the second is a system that can be applied to home devices for the monitoring of neovascular AMD, diabetic macular edema, and retinal vein occlusion [27,28].

The cost-effectiveness profile of monitoring patients at high risk of developing neovascular AMD is promising [29,30]. Home OCT for the follow up of patients with AMD may have benefits in maintaining visual acuity with a lower treatment burden, but it remains to be demonstrated [29,30].

### Future developments enabling personalized medicine

In future, some AI systems may be capable of segmenting an OCT image and linking the segmentation to real-world referral recommendations [31]. It has also been suggested that AI may be used to identify disease biomarkers and autonomously predict prognosis in a second step [32]. Future AI may also be capable of detecting anomalous regions, which can be used as novel marker candidates [32], as well as realizing a correlation between retinal morphology and visual function [33,34], to infer data such as visual acuity and microperimetry results.

The application of AI medical devices has been suggested for the management of patients affected by macular telangiectasia type 2. AI may be able to perform automated segmentation of retinal cavitations on OCT [35] and may also estimate retinal sensitivity and reproduce the results of clinical trial outcomes from OCT analysis [35,36].

Other authors have tried to evaluate the potential role of AI in the decision on optimal surgical timing and indication for epiretinal membranes. They proposed an automated method for the prediction of postoperative visual outcomes [37]. Postoperative anatomic changes may also be predicted by AI medical devices [38]. Furthermore, in patients with geographic atrophy, AI medical devices may be capable of associating the progression rate with morphologic and structural features. This would help identify patients who could benefit from future treatments [39] and also assist in evaluating the efficacy of novel drugs [40].

Future AI medical devices may help describe the progressive neurodegeneration underlying the process of retinal aging and may be capable of producing a risk score for AMD progression [41,42]. Visual acuity may be predicted from OCT features of patients affected by neovascular AMD, with the possibility of creating a personalized risk profile for each patient [20^▪^,^43^]. AI could also identify responders and nonresponders to treatment, and predict individual treatment needs [44,45] as well as the prognosis of patients affected by central serous chorioretinopathy, in order to individualize the treatment scheme. New biomarkers may be identified using heatmaps provided by the AI medical device [46], while other devices may enable us to better understand the role that the same biomarker, such as central retinal thickness or intraretinal fluid, has in different retinal pathologies [47].

#### Limitations of artificial intelligence and additional considerations

An increasing number of algorithms are being developed to improve the management of retinal diseases. Although prevention of vision loss is the ultimate goal, further studies are needed to evaluate the real clinical impact of the application of these algorithms in clinical practice [48].

Our literature search highlighted issues in the identification of AI medical devices in ophthalmology. Some of the search strategies described, although effective, require access to commercial databases that are not publicly available [49]. More transparency from regulators is required to allow for easy identification of AI medical devices. We also noted that almost all of the AI medical devices had undergone only retrospective studies at the time of their submission to regulatory authorities [50]. Depending on the strength of the studies, this may pose issues, including interpretation of the efficacy of AI medical devices in a real-life environment. Other potential issues are the heterogeneity of the regulation process in Europe and the United States, and the absence of a specific regulatory pathway [49].

The reference standard for AI plays a fundamental role in assessment of its performance. Reference standards are widely variable. It is important to test AI medical devices on independent data sets, but this testing phase is different for each device and the absence of a commonly used reference standard makes comparison challenging [51]. Moreover, external validation of AI medical devices described in the literature is rare and research on real-world applications is even rarer [35]. Therefore, before confirming the effectiveness of a predictive AI medical device, a prospective, thorough validation process needs to occur.

Notably, it is not always easy to understand the decision-making process made by AI, since there is not a simple relationship between input and output. In the analysis of medical imaging, heatmaps highlight the contribution of each image zone in the final decision process. However, they do not provide information on which exact features are considered relevant by the AI medical device [52]. Before regulators can approve AI medical devices for clinical use, evidence is needed to demonstrate that they are safe, effective, and inclusive [52]. Guidance provided by organizations such as the International Medical Device Regulators Forum and the Medicines and Healthcare products Regulatory Agency needs to be taken into consideration [53,54].

#### Use of artificial intelligence in everyday clinical practice

There are some prerequisites to the development of AI medical devices that can be applied to everyday clinical practice. Large developmental data sets with a significant number of abnormal cases are mandatory. Also, data sets used to measure final performance should have consensus ground truth from specialist clinicians to obtain a more reliable measure of the final predictive ability of the device [55]. Another issue that needs to be addressed to facilitate real-life application is health data poverty, which describes disparity in the quantity and/or quality of health data representing different individuals, groups, populations, but also different diseases. Data sets are the basis for the correct functioning of AI medical devices, and this may result in a device that is adequately effective for some populations but ineffective for others [56].

It has been highlighted that the quality of images provided to AI medical devices affects its discriminative ability. However, in real life, imaging is not standardized and reproducibility may be restricted [57]. The accuracy of AI medical devices may also be dependent on the imaging technology used, such as the camera make and model [58].

In addition, the accuracy drops when more than one disease is considered. Moreover, while the diagnostic accuracy is typically high, the predictive accuracy is usually lower. By using systemic parameters to develop multimodal AI medical devices, the predictive value of the algorithm may be improved [51,59,60]. Sensitivity and specificity thresholds need to be set at appropriate levels and a hybrid method, using both AI and human graders, may lead to the best results [51,59,60]. Decision curve analysis should be performed to evaluate the net benefit of the application of AI medical devices [61].

## VISION ACADEMY RECOMMENDATIONS

The Vision Academy has developed a series of recommendations addressing the benefits, limitations, and additional considerations of integrating AI into clinical practice.

### Recommendation 1: use of artificial intelligence in disease management

The Vision Academy recommends the application of AI medical devices to improve patient outcomes and alleviate treatment burden for both patients and healthcare providers. The technology should be cost effective, with additive value and synergy for the healthcare system.

These technologies should be implemented into everyday clinical practice at several points: screening [4], quantification of biomarkers to guide treatment, prognosis of retinal pathology, and prediction of treatment outcomes via anatomical and functional biomarkers.

### Recommendation 2: regulatory authorities and transparency

More transparency from regulatory authorities through publicly accessible databases is desired. Additionally, more prospective studies are needed for the validation of AI medical devices; it is necessary to test such devices on as many populations as possible, and randomized clinical trials remain the gold standard for assessing their efficacy. It would also be advisable to increase transparency around the composition of training; they should be representative of the overall population, of adequate quality and quantity, and accessible to regulators. The end user running each AI medical device and their degree of responsibility need to be specified, especially in the event of errors.

### Recommendation 3: nonretina specialist users and home monitoring

It is possible that AI medical devices for retinal disease management may be used in clinics without guidance from retinal specialists. It is therefore necessary to verify whether they can be translated into real-world deployment while maintaining high accuracy in clinical practice. Pathways for specialist referral and streamlined treatment need to be developed in parallel. Care pathways should ensure the capacity to follow up and treat an increasing number of patients. The workflow for the management of retinal diseases needs to adapt to new settings.

### Recommendation 4: sensitivity, specificity, and validation

AI medical devices should have high sensitivity and specificity thresholds and each algorithm in use should be validated. Validation and regulation should be defined according to international standards. Working parameters need to be well defined and the system should not be used outside these parameters.

Image data should be shared anonymously among various medical centers and countries, and such data sharing should be encouraged to overcome data poverty and ensure that AI medical devices are equally effective for all patient populations.

### Recommendation 5: legality and ethics

Ethical and legal ramifications should be thoroughly considered in the event the algorithm makes a mistake in diagnosis or prognosis of outcomes. Additionally, the degree of autonomy the AI medical device has in deciding the management of each patient should be discussed. In case of poor predicted outcomes, adequate communication with the patient should occur. The position of insurance companies regarding AI-based predictions should also be clarified.

## CONCLUSION

Due to its vast potential, AI is likely to lead to a profound change in clinical practice and in healthcare systems worldwide. As the models of care delivery change, there will be an increasing need for physicians to collaborate with broader teams, including technology experts and data scientists, to achieve the ultimate goal of sustainable ophthalmic services of universal quality. Before being used in clinical practice, the limitations of AI should be understood, AI medical devices should be validated by regulatory authorities, and the ethical and legal ramifications should be clarified.

Lastly, it is likely that the future of personalized medicine will be in prescriptive analytics. When AI medical devices are able to create personalized management pathways from a predictive analysis, this is likely to alter the existing predictive outcomes.

## Acknowledgements

*Editorial assistance was provided by Elle Lindsay, PhD, Macha Aldighieri, PhD, and Rebecca Fletcher, BA (Hons), of Complete HealthVizion, Ltd, an IPG Health Company, funded by Bayer Consumer Care AG, Pharmaceuticals Division, Basel, Switzerland.*

### Financial support and sponsorship

*The Vision Academy is a group of over 100 international ophthalmology experts who provide guidance for best clinical practice through their collective expertise in areas of controversy or with insufficient conclusive evidence. The Vision Academy is funded and facilitated by Bayer. The opinions and guidance of the Vision Academy outputs are those of its members and do not necessarily reflect the opinions of Bayer.*

*Financial arrangements of the authors with companies whose products may be related to the present report are listed in the ‘Conflicts of interest’ section, as declared by the authors.*

### Conflicts of interest

*Carla Danese is a consultant for Bayer. Tariq Aslam is a consultant for, and has received grants, speaker fees, and honoraria from, Allergan, Bayer Pharmaceuticals, Canon, NIHR, Roche, and Topcon. He is also a board member for the Vision Academy, Macular Society, and Fight for Sight charity. Jane Barratt has received honoraria from Bayer. Yu-Bai Chou is a consultant for Alcon and Bayer. Bora Eldem is a consultant for Allergan, Bayer, Novartis, and Roche. Nicole Eter is an advisor for AbbVie, Alcon, Apellis, Bayer, Biogen, Janssen, Novartis, and Roche and has received speaker fees from AbbVie, Apellis, Bayer, Novartis, and Roche and research grants from Bayer and Novartis. Richard Gale is a consultant for AbbVie, Allergan, Apellis, Bayer, Biogen, Boehringer Ingelheim, Notal, Novartis, Roche, and Santen and has received research grants from Bayer, Novartis, and Roche. Jean-François Korobelnik is a consultant for Allergan/AbbVie, Apellis, Bayer, Carl Zeiss Meditec, Janssen, Nano Retina, Roche, and Théa and is a member of the Data and Safety Monitoring Boards for Alexion and Novo Nordisk. Igor Kozak is a consultant for Alcon, Bayer, and Novartis. Paolo Lanzetta is a consultant for Aerie, AbbVie, Apellis, Bausch & Lomb, Bayer, Biogen, Boehringer Ingelheim, Genentech, Novartis, Ocular Therapeutix, Outlook Therapeutics, and Roche. Anat Loewenstein is a consultant for Allergan, Annexon, Bayer Healthcare, Beyeonics, Biogen, ForSight Labs, IQVIA, Iveric Bio, Johnson & Johnson, MJH Events, Nano Retina, Notal Vision, Novartis, Ocuphire Pharma, OcuTerra, OphtiMedRx, Roche, Ripple Therapeutics, Syneos, WebMD, and Xbrane. Paisan Ruamviboonsuk is a consultant for, and has received research funds from, Bayer and Roche. Taiji Sakamoto is a consultant for Bayer Yakuhin, Boehringer Ingelheim, Chugai, Nidek, Nikon, Novartis, Santen, and Senju. Daniel S.W. Ting has received research grants from the National Medical Research Council Singapore, Duke-NUS Medical School Singapore, and Agency for Science, Technology and Research Singapore. Peter van Wijngaarden is the co-founder of Enlighten Imaging, an early-stage medical technology start-up company devoted to hyperspectral retinal imaging and image analysis, including the development of AI systems, and has received research grant support from Bayer and Roche and honoraria from Bayer, Mylan, Novartis, and Roche. Sebastian M. Waldstein is a consultant for Apellis, Bayer, Boehringer Ingelheim, Novartis, Roche, and Santen. David Wong is a consultant for AbbVie, Alcon, Apellis, Bayer, Bausch Health, Biogen, Boehringer Ingelheim, Novartis, Ripple Therapeutics, Roche, Topcon, and Zeiss, has received financial support (to institution) from Bayer, Novartis, and Roche, and is an equity owner at ArcticDx. Lihteh Wu is a consultant for Bayer, Lumibird Medical, Novartis, and Roche. Miguel A. Zapata is a consultant for Novartis and Roche, has received grants and speaker fees from DORC, Novartis, and Roche, honoraria from Alcon, Bayer, DORC, Novartis, and Roche, has served on advisory boards for Novartis and Roche, has received equipment from Allergan, and has stock or stock options in UpRetina. Javier Zarranz-Ventura has received grants from AbbVie, Allergan, Bayer, Novartis, and Roche, has served on scientific advisory boards for AbbVie, Allergan, Bayer, Novartis, and Roche, and has been a speaker for AbbVie, Alcon, Alimera Sciences, Allergan, Bausch & Lomb, Bayer, Brill Pharma, DORC, Esteve, Novartis, Roche, Topcon Healthcare, and Zeiss. Aditya U. Kale, Xiaorong Li, and Xiaoxin Li have no conflicts of interest to report.*

## REFERENCES AND RECOMMENDED READING

Papers of particular interest, published within the annual period of review, have been highlighted as:

▪ of special interest
▪▪ of outstanding interest
